# Rolling-induced Face Centered Cubic Titanium in Hexagonal Close Packed Titanium at Room Temperature

**DOI:** 10.1038/srep24370

**Published:** 2016-04-12

**Authors:** H. C. Wu, A. Kumar, J. Wang, X. F. Bi, C. N. Tomé, Z. Zhang, S. X. Mao

**Affiliations:** 1 School of Materials Science and Engineering, Beihang University, Beijing 100191, People’s Republic of China; 2 Materials Science and Technology Division, Los Alamos National Laboratory, Los Alamos, NM 87545, USA; 3 Mechanical and Materials Engineering, University of Nebraska-Lincoln, Lincoln, NE 68588, USA; 4 Department of Materials Science, State Key Lab of Si Materials, ZheJiang University, Hangzhou, Zhejiang, China; 5 Department of Mechanical Engineering and Materials Science, University of Pittsburgh, Pittsburgh, Pennsylvania 15261, USA

## Abstract

Combining transmission electron microscopes and density functional theory calculations, we report the nucleation and growth mechanisms of room temperature rolling induced face-centered cubic titanium (fcc-Ti) in polycrystalline hexagonal close packed titanium (hcp-Ti). Fcc-Ti and hcp-Ti take the orientation relation: 〈0001〉_hcp_||〈001〉_fcc_ and 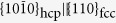, different from the conventional one. The nucleation of fcc-Ti is accomplished via pure-shuffle mechanism with a minimum stable thickness of three atomic layers, and the growth via shear-shuffle mechanisms through gliding two-layer disconnections or pure-shuffle mechanisms through gliding four-layer disconnections. Such phase transformation offers an additional plastic deformation mode comparable to twinning.

When metals and alloys are subject to severe plastic deformation, phase transformation could be activated to accommodate external strain^1^,^2^,^3^,^4^,^5^. For metals and alloys with hexagonal close-packed (hcp) structure, twinning is commonly triggered because of insufficient number of slip systems^6^. In addition to deformation twinning, the hexagonal close-packed structure to cubic structure transformation has been observed in cobalt^7^,^8^, Co-32%Ni alloy^9^,^10^, Ti-based alloys^11^ and InAs nanowires^12^, and acts as an additional plastic deformation mechanism comparable to twinning. The orientation relation between the two phases has been characterized to be 〈11
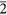
0〉_hcp_||〈110〉_fcc_ and {0001}_hcp_||{111}_fcc_. The hcp-fcc transformation can be accomplished via the gliding of Shockley partial dislocations on every two hexagonal close packed planes, resulting in the stacking sequence change of hexagonal close packed planes from …ABABAB… to …ABCABC….. With respect to the combination of Shockley partial dislocations, macroscopic strain will be resulted accompanying with phase transformation when all Shockley partials have the same Burgers vector, or, the transformation will not generate macroscopic strain, so-called zero-strain transformation^12^,^13^,^14^, when the net Burgers vector of all Shockley partials is equal to zero.

Using TEM, we observed rolling-induced fcc-Ti bands at room temperature in hcp-Ti polycrystalline aggregates. Such phase transformation offers an additional plastic deformation mode comparable to twinning. Surprisingly, the fcc-Ti and the hcp-Ti take the orientation relation: {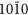}_hcp_||{110}_fcc_ and 〈0001〉_hcp_||〈001〉_fcc_, different from the well-known fcc-hcp orientation relation. It is known that pure Ti has the hexagonal closed packed (hcp) structure at ambient temperatures and body centered cubic (bcc) structure at elevated temperatures, while fcc phase does not exist in the equilibrium phase diagram^15^,^16^. By combining topological model, density functional theory (DFT) calculations, and microscopes, we identified the nucleation and growth mechanisms of fcc-Ti in hcp-Ti. We found the nucleation via pure-shuffle and the growth via shear-shuffle mechanisms, different from the established phase transition path via the collective gliding of Shockley partial dislocations on hexagonal close packed planes.

## Results

**Figure 1:**
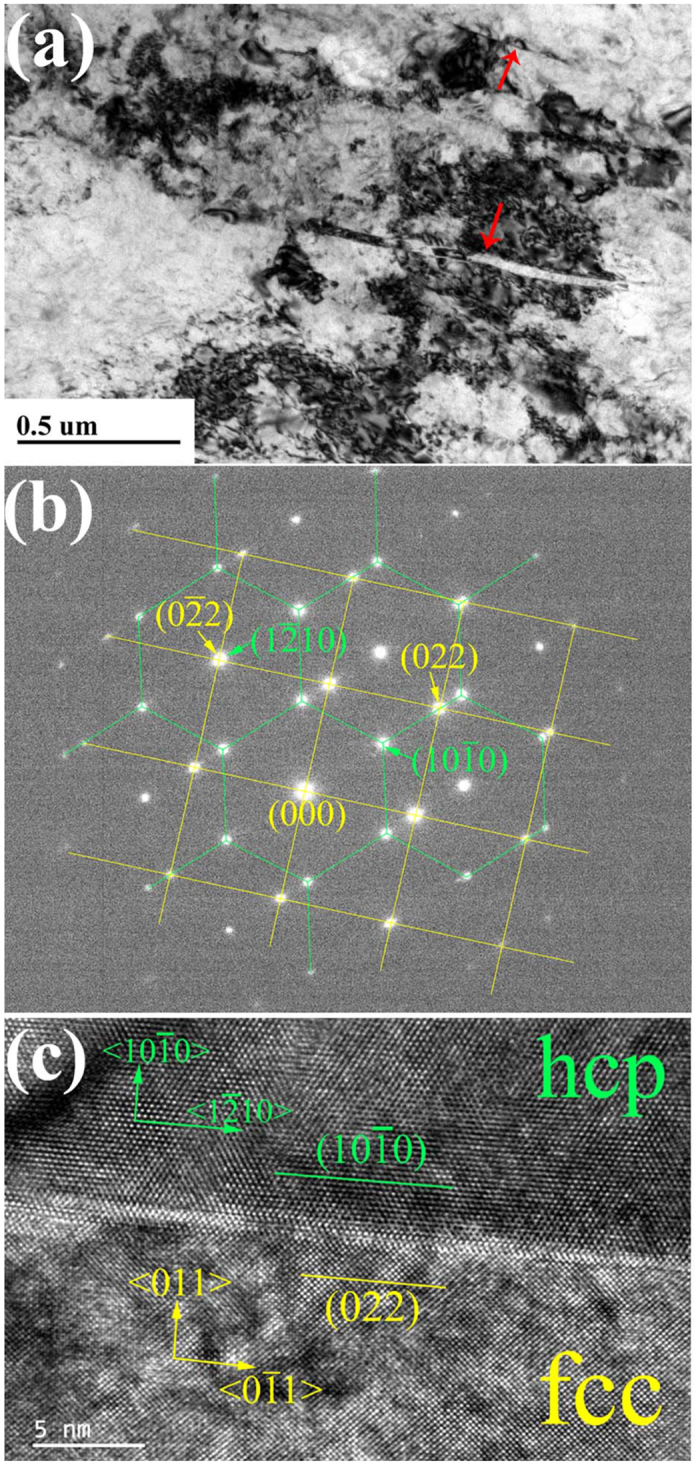
Orientation relationship between hcp matrix and fcc band. (**a**) Platelet bands in cold-rolled titanium indicated by red arrows. (**b**) SAED patterns of the interface between hcp and fcc, where patterns outlined by green lines corresponding to hcp and by yellow lines corresponding to fcc. (**c**) HRTEM image of the interface showing the orientation relationship to be 〈0001〉_hcp_||〈001〉_fcc_ and {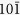
0}_hcp_||{011}_fcc_.

Figure 1a shows typical TEM micrograph of the cold-rolled Ti specimen. Platelet bands with 5 nm to 30 nm in thickness were observed in the hcp-Ti matrix after the sample underwent a total rolling reduction of 50% in thickness, as indicated in Fig. 1 by red arrows. Selected area electron diffraction (SAED) in Fig. 1b and HRTEM analysis in Fig. 1c reveals an fcc crystal structure of these bands (details can be found in Fig. S1). The SAED pattern in Fig. 1b reveals that the orientation relation is 〈0001〉_hcp_||〈001〉_fcc_ and {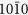}_hcp_||{110}_fcc_, being consistent with the HRTEM image in Fig. 1c. This orientation relation is different from the conventional one (〈11
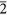
0〉_hcp_||〈011〉_fcc_ and {0001}_hcp_||{111}_fcc_), indicating that the corresponding phase transformation can’t be accomplished via successive glide of Shockley partial dislocations on hexagonal close packed planes. In what followed, we addressed nucleation and growth mechanisms of fcc-Ti in hcp-Ti matrix.

**Figure 2:**
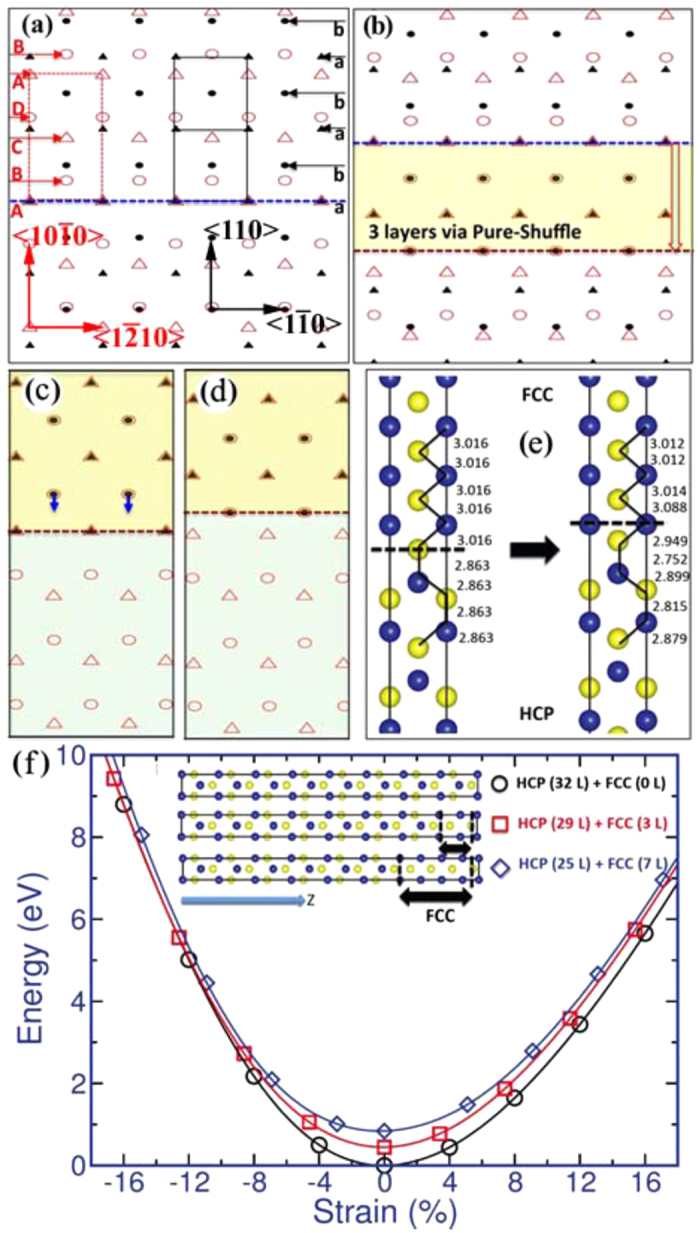
Topological models and DFT calculations: (**a**) Coherent dichromatic complex of hcp-Ti and fcc-Ti where the two crystals retain coherency in the x- and z- axes and adopt the orientation relation described as the x-axis along the 〈
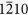
〉_hcp_ and 〈
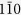
〉_fcc_, the y-axis along 〈
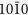
〉hcp and 〈110〉fcc, and the z-axis along 〈0001〉hcp and 〈001〉fcc. (**b**) Nucleation of an fcc band with three atomic layers in thickness via pure-shuffle mechanism. (**c**,**d**) Two possible types of coherent phase interface. (**e**) Relaxation of interface in (**c**) to interface in (**d**), indicating only interface in (**d**) is energetically stable. (**f**) DFT calculations of fcc-Ti band embedded in hcp matrix to exam stability of fcc bands with respect to external strain.

Figure 2a shows coherent dichromatic complex of hcp-Ti and fcc-Ti where the two crystals retain coherence in the x- and z- axes and the x-axis is along the 〈
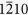
〉_hcp_ and 〈
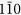
〉_fcc_, the y-axis along 〈
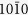
〉_hcp_ and 〈110〉_fcc_, and the z-axis along 〈0001〉_hcp_ and 〈001〉_fcc_, respectively. The stacking sequence of {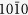} plane is …ABCD…. The interplanar spacing between BC or DA is double of that between AB or CD. The stacking sequence of {110} plane is …abab… and the interplanar spacing between ab and ba is the same. This crystallographic difference raises three following questions: first, how many types of interface structure may form corresponding to the different stacking sequences of their atomic planes? Second, is there a minimum thickness for a stable fcc-Ti band? Third, how does an fcc-Ti band form and grow?

To address the above three questions, we performed topological analysis^17^,^18^,^19^. Disregarding of kinetics and energetics consideration, an fcc-Ti band may nucleate with different atomic thickness, comprising two, three, four, or even more atomic planes. The possible structures associated with nucleation of an fcc-Ti band are shown in Figs S2–S5. Topological analysis reveals that a two-layer fcc-Ti band (Fig. S3) may form via shear-shuffle mechanism^20^; a three-layer fcc-Ti band (Fig. S4) may form via either shear-shuffle^20^ or pure-shuffle mechanisms^21^; a four-layer fcc-Ti band (Fig. S5) may form via either pure-shuffle^21^ or shear-shuffle mechanisms by two steps using the two-layer shear-shuffle mechanism^20^. It should be noticed that shear-shuffle is accomplished via the cooperation of gliding a two-layer disconnection between the plane of BC or DA that has the larger interplanar spacing. The Burgers vector of the two-layer disconnection is equal to 1/6〈
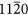
〉.

Among all possible fcc-band structures, topological analysis reveals two types of coherent phase interface (Fig. 2c,d). We further performed DFT calculations^22^,^23^,^24^ to examine structures of fcc-Ti, hcp-Ti and fcc-Ti/hcp-Ti interfaces. 19 × 19 × 11 and 19 × 19 × 19 Γ-centered Monkhorst Pack k-point meshs for the integration of primitive hexagonal Brillouin zone (BZ) are used in determining lattice constants for fcc and hcp structures, respectively. The optimized lattice constants for hcp-Ti are a = 0.2924 nm, c = 0.4625 nm with c/a = 1.582, which are in good agreement with the experimental lattice constants. The optimized lattice constant for the fcc-Ti is equal to 0.4089 nm. We then examined the two interfaces with a computational cell containing 48 atoms. A Γ-centered Monkhorst-Pack k-point mesh of 19 × 11 × 5 was used in these simulations. Figure 2e shows atomic structures in our DFT calculation: initial structure of the interface in the left (same as Fig. 2c, and contains one unit length in the x and z-directions) and the relaxed structure of the interface in the right (same as Fig. 2d). The position of interface plane was identified according to the bond length and angle. Corresponding to the periodic boundaries in the x and z directions, the identification can be simplified to test the change in the interplanar spacing. The black dashed line indicates the shared interface plane between fcc and hcp structures. After relaxation, the interplanar spacing decreases from 0.3088 nm to 0.2949 nm, indicating the shared interface plane move upward one atomic plane. Thus, only one type of interface in Fig. 2d is energetically stable. The interface formation energy is 9.6 mJ/m^2^, much smaller than that of coherent twin boundaries (297 mJ/m^2^ and 499 mJ/m^2^ for extension twins {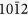}〈
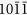
〉 and {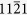}〈
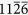
〉 and 75.3 mJ/m^2^ and 395 mJ/m^2^ for compression twins {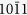}〈
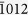
〉 and {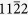}〈11
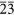
〉).

Giving the answer to the second question from DFT calculations, a minimum fcc-Ti band may have three atomic layers in thickness (Fig. 2b), which is surrounded by two identical, stable interfaces. Such three-layer fcc-Ti band nucleates via pure-shuffle mechanism (Fig. S4c).

We further studied the stability of fcc-Ti bands that are embedded in hcp-Ti matrix. Three computational cells containing the same number of Ti atoms are constructed for comparison (Fig. 2f). Case 1 is a pure hcp structure containing 32 atomic layers, case 2 comprises 29 atomic layers hcp structure and 3 atomic layers fcc structure (referred to be 3L-FCC structure), and case 3 is consisting of 25 atomic layers hcp structure and 7 atomic layers fcc structure (referred to be 7L-FCC structure). Cases 2 and 3 have two identical hcp-fcc interfaces. Three-dimensional periodic boundaries are adopted in all simulations. Under uniaxial tensile loading, the 3L-FCC and 7L-FCC structures are stable. Interestingly, the pure hcp structure at a strain of 4% has higher potential energy than the 3L-FCC structure at zero strain, and at a strain of 5.5% has higher potential energy than the 7L-FCC structure at zero strain. This indicates that phase transformation could commence at these strains with the assistance of thermal fluctuation. Under uniaxial compressive loading, the 3L-FCC structure recovers into a pure hcp structure through atomic shuffle at the strain greater than 10%. Thus, a minimum, stable fcc band has three atomic layers thick and can be nucleated at a tension strain above 4%.

**Figure 3:**
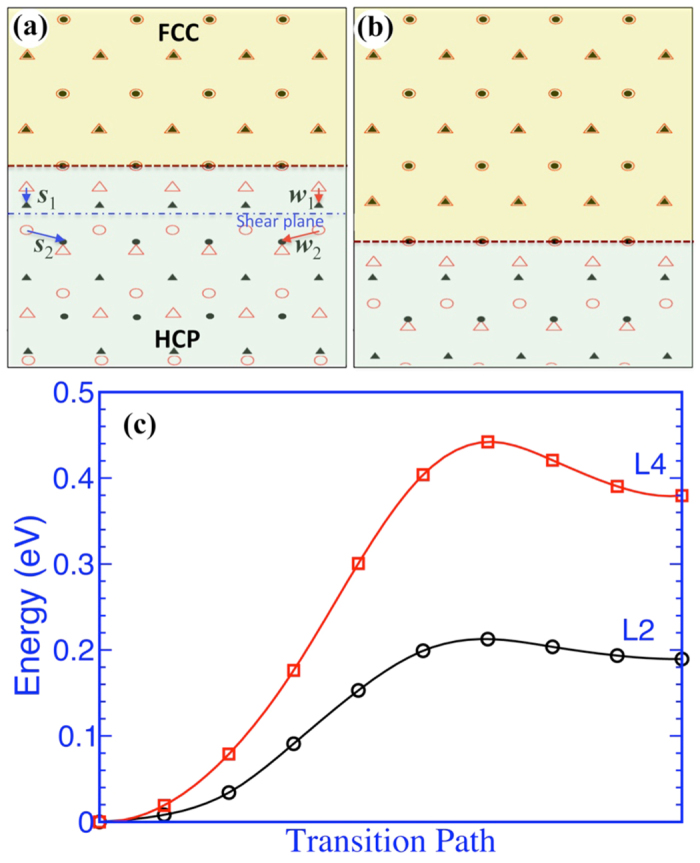
(**a**,**b**) Topological models illustrating growth of fcc band. Two sets of displacements, *s*_1_ and *w*_1_, and *s*_2_ and *w*_2_, correspond to two opposite-signed two-layer disconnections (shear-shuffle). (**c**) The change in the potential energy with respect to the transition path. L2 represents a two-layer shear-shuffle and L4 represents a four-layer pure-shuffle. The two crystals adopt the orientation relation described as the x-axis along the 〈
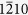
〉_hcp_ and 〈
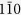
〉_fcc_, the y-axis along 〈
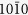
〉hcp and 〈110〉fcc, and the z-axis along 〈0001〉hcp and 〈001〉fcc.

Once a finite thick fcc band forms in hcp matrix, growth of fcc band is associated with migration of coherent phase interface. Since there is only one energetically stable interface, migration of phase interface must involve even numbers of atomic layers as a unit in order to form a new stable interface. Figure 3a shows shear-shuffle mechanisms. The shear displacement is equal to 1/2*a* of hcp structure, corresponding to the glide of a 2-layer disconnection^20^ with the Burgers vector 1/6〈
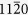
〉 (equal to the projection of the displacement *s*_2_ or *w*_2_ on the glide plane in Fig. 3a). The glide plane is in-between the planes BC or DA that has the larger interplanar spacing. Accompanying with the shear, atoms in the two layers also experience a shuffle displacement *s*_1_ (equal to *w*_1_). After a two-layer growth, the new interface is identical to the initial interface (Fig. 3b). Thus, fcc-Ti can grow via a shear-shuffle mechanism with a repeatable operation by every two atomic layers. There are two scenarios associated with the growth of an fcc-Ti band. Under a shear stress parallel to the interface plane, interface migrates via the shear-shuffle mechanism with the same-signed disconnections, resulting in a macro-scale shear strain and a normal tension strain. Under a tension stress normal to the interface plane, interface migrates via the collective glide of two opposite-signed disconnections, resulting in zero macro-scale shear strain and a normal tension strain. In other words, the growth of a fcc-Ti band under normal tension stress can be accomplished via pure-shuffle mechanism with four atomic layers as one unit. Using nudged elastic band method^25^ (Fig. 3c), we perform DFT calculations to compute the energy profile with respect to the shear-shuffle by two layers and the pure-shuffle by four layers. The shear-shuffle mechanism experiences a smaller energy barrier than the pure-shuffle mechanism.

**Figure 4:**
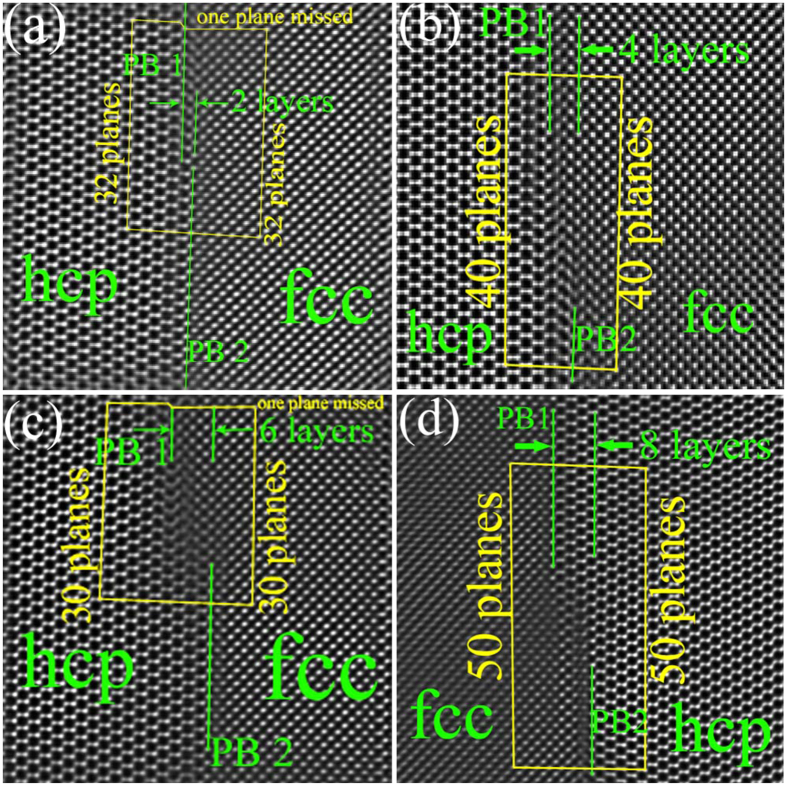
Inversed fast Fourier transformation (IFFT) images of 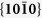
_hcp_||{110}_fcc_ interface taken by Cs-corrected HRTEM. Phase boundaries (PB) are marked in green lines and Burgers circuits in yellow lines. (**a**) a two-layer step, (**b**) a four-layer step, (**c**) a six-layer step, (**d**) an eight-layer step. The original images corresponding to (**a–d**) are in Fig. S6.

To examine our predictions about the atomic structure of the stable interface and the corresponding migration mechanism of phase interface, we characterized structural features of phase interfaces with the focus on steps along phase boundaries using Cs-corrected HRTEM (Fig. S6) and corresponding inversed fast Fourier transformation (IFFT) (Fig. 4), where steps along the boundary can be viewed as a trace associated with boundary migration^26^,^27^,^28^. First, the energetically stable interface (Fig. 2d) predicted using DFT calculations is evidenced in all Cs-corrected HRTEM images. A magnified image for comparison is also provided in Supplementary (Fig. S7). Second, we observed four types of steps: two-layer (Fig. 4a), four-layer (Fig. 4b), six-layer (Fig. 4c) and eight-layer (Fig. 4d), hinting that two atomic layers may serve as a minimum migration unit.

The Burgers vector of these steps/disconnections was characterized by drawing Burgers circuits in Cs-corrected HRTEM images. Figure 4a shows that the 2-layer step (or disconnection) has the shear displacement of 1/6〈
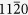
〉, in agreement with the shear-shuffle model in Fig. 3a. For the 4-layer step (or disconnection, Fig. 4b), Burgers circuit analysis shows that there is no extra plane along the interface, meaning that the net shear displacement associated with the 4-layer step is zero. This indicates that the 4-layer step may form via either four-layer pure-shuffle mechanism or two-layer opposite-signed shear-shuffle mechanisms. In order to clarify this, a 6-layer step is further analyzed. Figure 4c shows that the 6-layer step (or disconnection) is associated with one extra plane along the interface, corresponding to a net shear displacement of 1/6〈
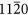
〉. If the 6-layer step is formed via successive glide of three same-signed two-layer disconnections, the number of extra planes in fcc structure should be three. Thus, the 6-layer step must form via alternative glide of two-layer disconnections with the opposite-signed shear displacement. It is also possible that the 6-layer step is formed via one four-layer pure-shuffle plus one two-layer shear-shuffle, while the pure-shuffle process is operated by two opposite-signed two-layer shear shuffle. This is further confirmed by analyzing an 8-layer step (Fig. 4d) where Burgers circuit analysis shows that the net shear displacement around the step is also zero. Thus, the growth of an fcc-Ti band can be rationalized as follows. Under a complicated local stress condition, if a shear stress acts on the interface or there is a thermal fluctuation, nucleation and glide of one two-layer disconnection is kinetically favored, resulting in the drop of the local shear stress^29^. Then the opposite-signed two-layer disconnection operates, achieving a four-layer pure-shuffle migration^20^,^21^. The two 2-layer steps/disconnections attract each other, and will glide together.

We so far proposed and examined nucleation and growth mechanisms of fcc-Ti bands in hcp-Ti matrix. Recently, Hong *et al*. observed formation of fcc-Ti bands in hcp-Ti matrix and proposed a growth model based on successive glide of Shockley partial dislocations (with Burgers vector of 1/6〈11
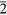
0〉) on prismatic planes^2^. We would like to point out that such a model seems not well justified. Firstly, the gliding of Shockley partial dislocations does not generate the lattice expansion normal to the phase boundary (corresponding to macroscopic transformation strain). Second, the successive gliding of Shockley partial dislocations with the same Burgers vector will result in localized shear deformation parallel to the phase boundary. Thus, a sharp step is not energetically favored. Thirdly, a sharp step can form if these partial dislocations have the opposite sign. However the question is how to change the sign of shear stress on the phase boundary on every two atomic planes. In addition, for polycrystalline aggregates with fcc bands formed entirely in the interior of grains, there is no effective positions such as free surface, grain boundary or crack tip for Shockley partial dislocations nucleation. Thus, the Shockley partial dislocation model cannot serve as an available explanation for the phase transformation in polycrystalline titanium aggregates.

## Discussion

Such hcp-to-fcc phase transformation via pure-shuffle nucleation and shear-shuffle growth mechanisms offers an additional plastic deformation mode comparable to compression twins. The c/a ratio of Ti (1.58) is markedly less than the ideal value of 1.63 for hcp structures and Ti can plastically deform via multiple twinning modes including {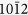}, {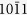}, {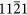} and {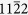}^30^,^31^. However, we only observed a few of {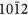} extension twins after rolling at room temperature, although the total deformation reaches up to 50% after three rolling passes. For hcp metals, rolling can develop a strong basal texture^30^,^31^,^32^,^33^. Therefore, most grains are subjected to an effectively compressive stress along the c-axis and a tensile stress normal to their prismatic planes. Twin modes of {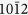}〈
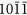
〉 and {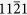}〈
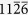
〉 can be activated under an effective tensile stress along the c-axis, resulting in a tensile strain normal to basal plane and a compressive strain normal to prismatic plane, while twin modes of {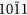}〈
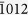
〉 and {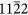}〈11
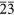
〉 can be activated under an effective compressive stress along the c-axis, resulting in a compressive strain normal to basal plane and a tensile strain normal to prismatic plane. Therefore, a few of extension twins observed in our samples can be ascribed to the small diversity of grain orientations corresponding to a strong basal texture. Intriguingly, compression twins presumably activated were not observed in our samples. Instead, stress-induced phase transformation is activated to accommodate plastic deformation. During phase transformation, fcc {220} planes are transformed from hcp {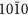} planes where one corrugated {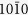} plane will split into two {220} planes. The interplanar spacing of {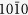} plane is 0.2532 nm and {220} plane is 0.1446 nm, and there is a lattice expansion of 14.2% normal to the prismatic plane. Thus the hcp-to-fcc phase transformation can accommodate macro strain comparable to compression twins. Nevertheless, the fact that compression twins are suppressed but phase transformation is favored needs to be clarified in the future.

Although the influence of fcc bands on the mechanical properties of bulk Ti needs further exploration, it can be deduced that fcc bands may improve materials strength. Corresponding to interface crystallography, the easy gliding basal 〈a〉 slip in matrix can be effectively blocked at the phase interface due to the discontinuity of slip systems across the interface. This point is confirmed by a recent study of Hong *et al*. in which the room-temperature yield strength of Ti polycrystalline were observed to increase from 381 MPa in the as-received state to 731 MPa after fcc bands formed^2^.

In summary, we observed rolling-induced hcp to fcc phase transformation in polycrystalline commercially pure titanium at room temperature. The orientation relation between hcp and fcc is 〈0001〉hcp||〈001〉fcc and {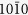}hcp||{110}fcc. The nucleation of fcc-Ti band is accomplished via pure-shuffle mechanism with a minimum stable thickness of three atomic layers, and the migration of phase boundary is accomplished via shear-shuffle mechanisms through the successive glide of opposite-signed disconnections with a minimum thickness of two atomic layers or pure-shuffle mechanisms with a minimum thickness of four atomic layers. Cs-corrected HRTEM analysis of steps along the phase boundary further confirmed structural characters of phase boundary and growth mechanisms of fcc-Ti bands in which two atomic layers serve as a minimum growth unit. Such phase transformation offers an additional plastic deformation mode comparable to compression twins.

## Methods

Polycrystalline commercially-pure titanium plates with thickness of 200 um were used for the experiment. The plates were annealed at 1250 ^o^C for 3 hours, and then room temperature rolling was conducted for three passes to a total reduction of 50% in thickness. The rolled samples were prepared for TEM characterization by double-jet electrolytic polishing at −25 ^o^C in electrolyte consisting of 6% perchloric acid and 94% alcohol. TEM observation and interface characterization were conducted on a JEM2100F electron microscope operated at 200 kV. The phase boundary characterization was conducted on a Titan G^2^ 60–300 Cs-corrected electron microscope operated at 300 kV.

### Density-Functional Theory Calculations

We used generalized gradient approximation (GGA) for the exchange correlation functional with the Perdew-Becke-Erzenhof (PBE)^34^ parameterization, and PAW pseudopotentials for the interaction between valence electrons and ionic cores^35^,^36^. The Ti pseudopotential that we used in our calculations includes 4 valence electrons (4s2, 3d2)^37^. We used a plane wave cutoff of 500 eV. The number of k-points varies with each calculation as defined in the main text. An optimized structure was obtained when the force on each atom is smaller than 0.0001 eV/nm.

## Additional Information

**How to cite this article**: Wu, H. C. *et al*. Rolling-induced Face Centered Cubic Titanium in Hexagonal Close Packed Titanium at Room Temperature. *Sci. Rep*. **6**, 24370; doi: 10.1038/srep24370 (2016).

## Supplementary Material

Supplementary Information
