# Supplementary material for: Rolling-induced Face Centered Cubic Titanium in Hexagonal Close Packed Titanium at Room Temperature
Source: Sci Rep. 2016 Apr 12;6:24370. doi: 10.1038/srep24370 (PMC4828854; doi:10.1038/srep24370)
Supplement: Supplementary Information [file srep24370-s1.pdf]

Supplementary Information

Rolling-induced Face Centered Cubic Titanium in Hexagonal Close Packed Titanium at Room Temperature

H. C. Wu[1,a], A. Kumar[2,a], J. Wang[3,*], X. F. Bi[1,*], C. N. Tomé[2], Z. Zhang[4] and S.X. Mao[5,*]

[1]School of Materials Science and Engineering, Beihang University, Beijing 100191, People's Republic of China
[2]Materials Science and Technology Division, Los Alamos National Laboratory, Los Alamos, NM 87545, USA
[3]Mechanical and Materials Engineering, University of Nebraska-Lincoln, Lincoln, NE 68588, USA
[4]Department of Materials Science, State Key Lab of Si Materials, ZheJiang University, Hangzhou, Zhejiang, China
[5]Department of Mechanical Engineering and Materials Science, University of Pittsburgh, Pittsburgh, Pennsylvania 15261, USA

[a]These authors contributed equally to this work.
[*]Corresponding authors: sxm2@pitt.edu (SXM); bixf@buaa.edu.cn (XFB); jianwang@unl.edu (JW)

Figure S1. Structural characterization of fcc-Ti.
Figures S2 to S5. Topological Analysis.
Figure S6. Original images of Cs-corrected HRTEM.
Figure S7. Magnified images of the stable hcp/fcc interface.

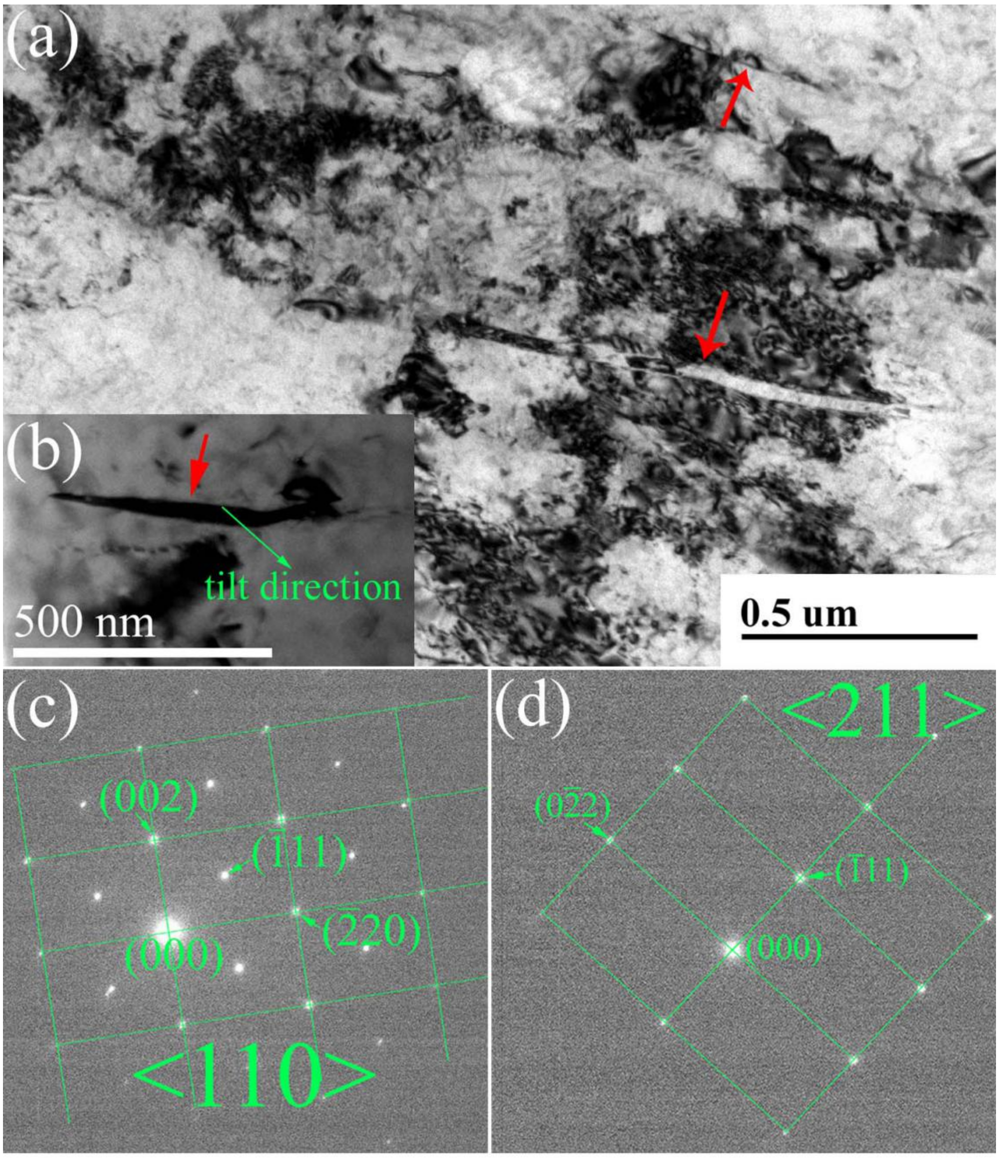

Figure S1. Structural characterization of fcc-Ti: (a) Platelet bands indicated by red arrows in hcp-Ti. (b) A magnified image of a platelet band. (c) SAED patterns of the band in (b) along a certain zone axis, which matches with fcc <110>. (d) SAED patterns of the band in (b) along a certain zone axis, which matches with fcc <112>. The tilt angle between the two observed directions (b and c) is 29°, close to the ideal tilt angle 30° in an fcc structure.

Topological Analysis of nucleation of fcc-Ti band in hcp matrix

Figures S2-S5 show coherent dichromatic complex of hcp-Ti (red symbols) and fcc-Ti (black symbols) where the two crystals retain coherency in the x- and z- axis and adopt the orientation relation designated as the x-axis along the $<1\bar{2}10>_{hcp}$ and $<1\bar{1}0>_{fcc}$, the y-axis along $<10\bar{1}0>_{hcp}$ and $<110>_{fcc}$, and the z-axis along $<0001>_{hcp}$ and $<001>_{fcc}$, respectively. Circle and triangle symbols represent atomic planes along the z-axis. The x-axis is the horizontal axis, the y-axis is the vertical axis, and the z-axis is pointing out the paper.

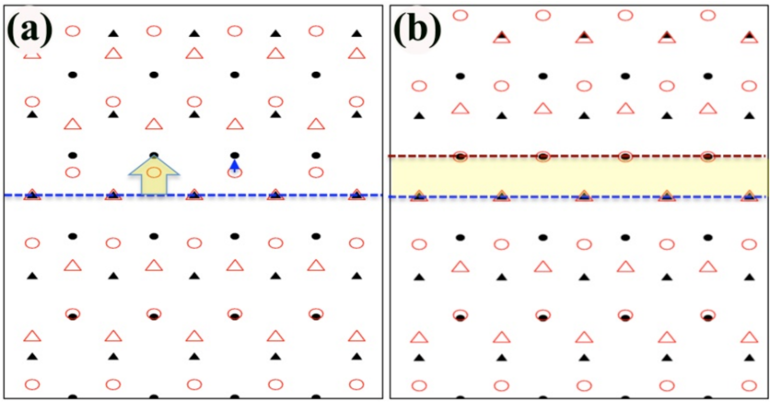

Figure S2. Nucleation of a one-layer fcc-Ti via pure shuffle mechanism. (a) Atoms movement mode, which only involves a small shuffle displacement. (b) A one-layer fcc-Ti nucleus in hcp-Ti matrix and two phase boundaries indicated by dashed lines. the arrow indicates the growth direction.

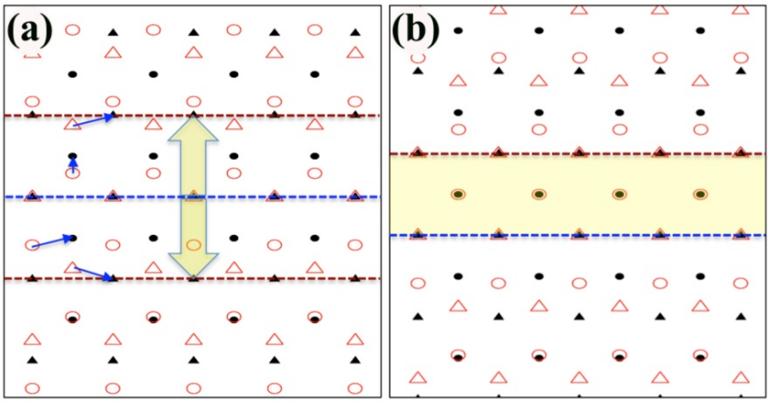

Figure S3. Nucleation of a two-layer fcc-Ti band via shear-shuffle mechanism. (a) Atoms movement mode, which involves both shuffle and shear components. (b) A two-layer fcc-Ti nucleus in hcp matrix and the two phase boundaries are of different type.

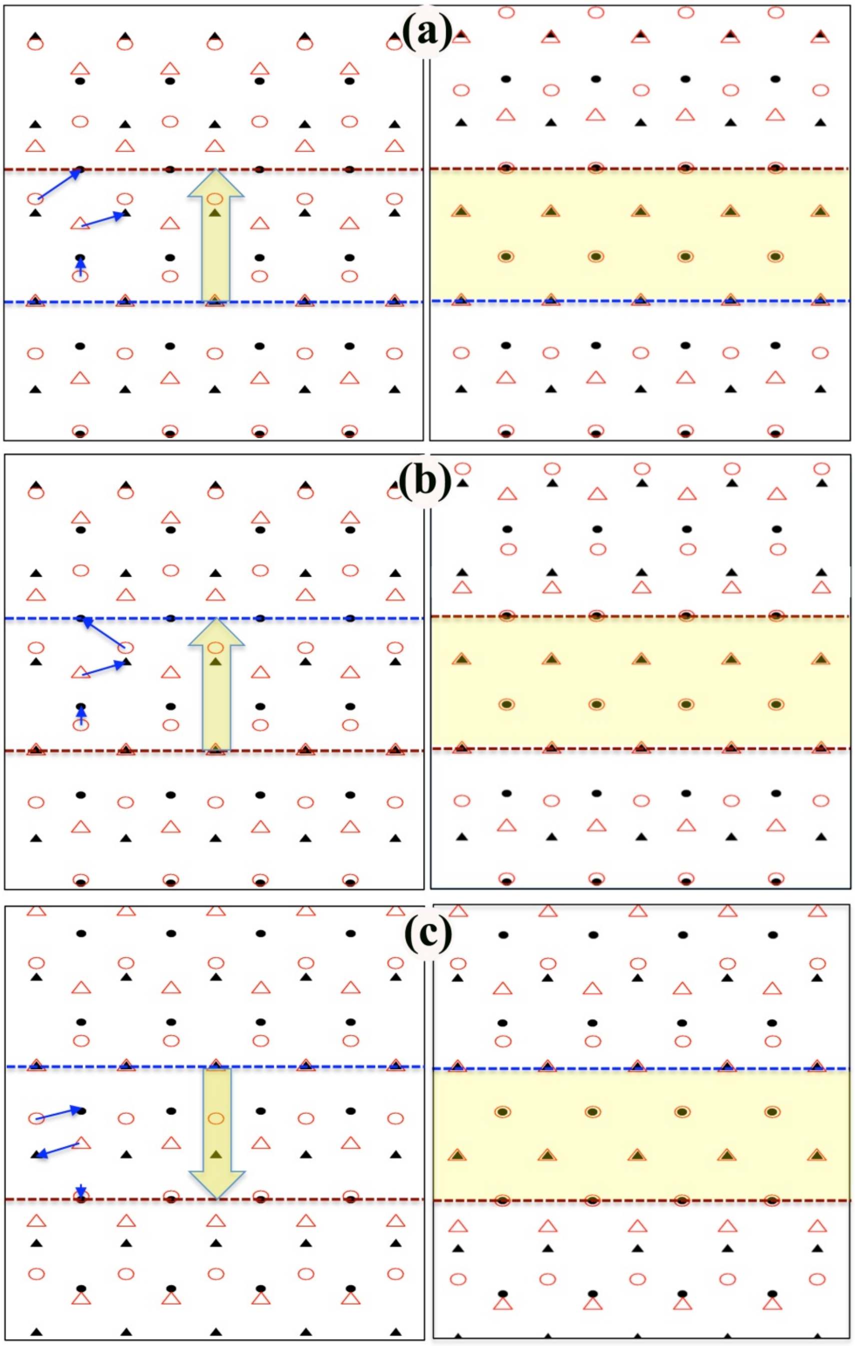

Figure S4. Nucleation of a three-layer fcc-Ti band via different mechanisms. (a) Shear-shuffle mechanism, forming two identical phase boundaries. (b) Shear-shuffle mechanism, and the two phase boundaries are of different type. (c) Pure-shuffle mechanism, via which two identical phase boundaries differing from that shown in (a) form.

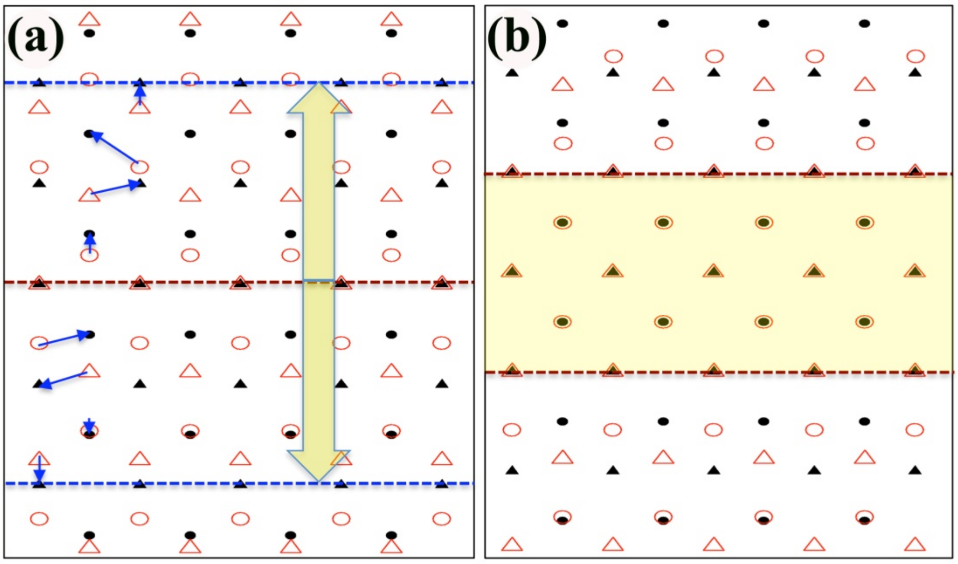

Figure S5. Nucleation of a four-layer fcc-Ti band via pure shuffle mechanism. (a) Atoms movement mode, either upward or downward is an effective approach. (b) A four-layer fcc-Ti in hcp matrix, and the two phase boundaries are of different type.

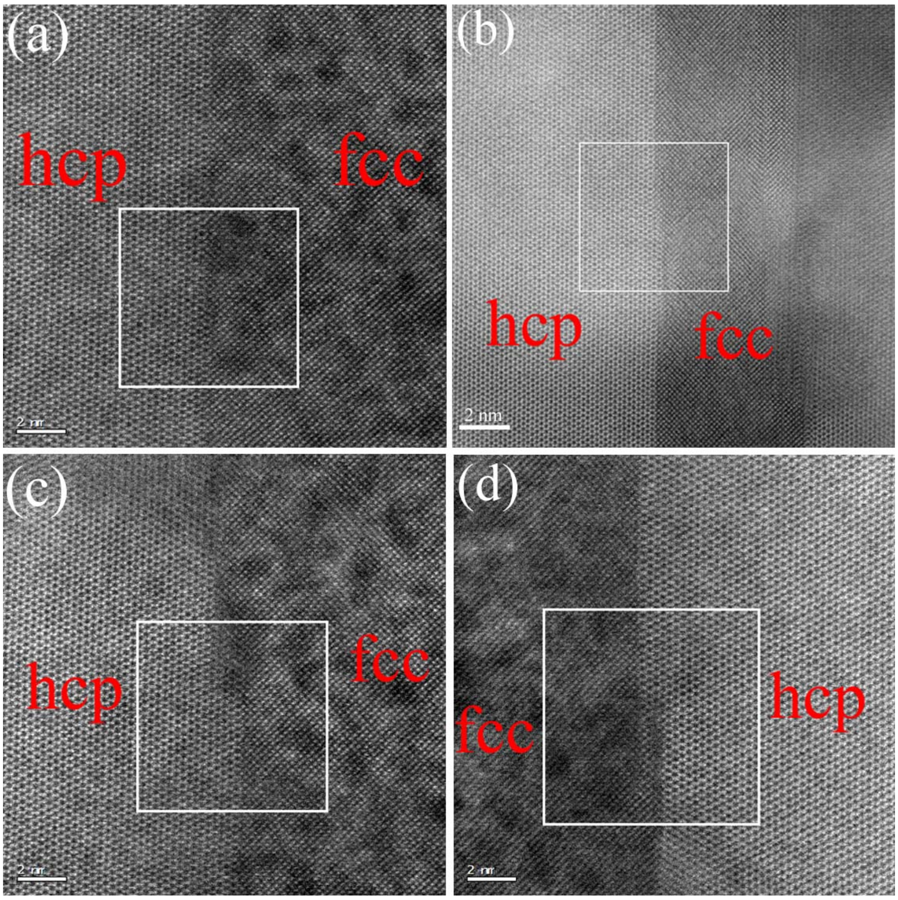

Figure S6. Cs-corrected HRTEM of $\{10\bar{1}0\}_{hcp}||\{110\}_{fcc}$ interfaces, (a) A two-layer step, (b) a four-layer step, (c) a six-layer step, and (d) an eight-layer step. The region outlined by the white box is corresponding to Fig. 4a-d in the text.

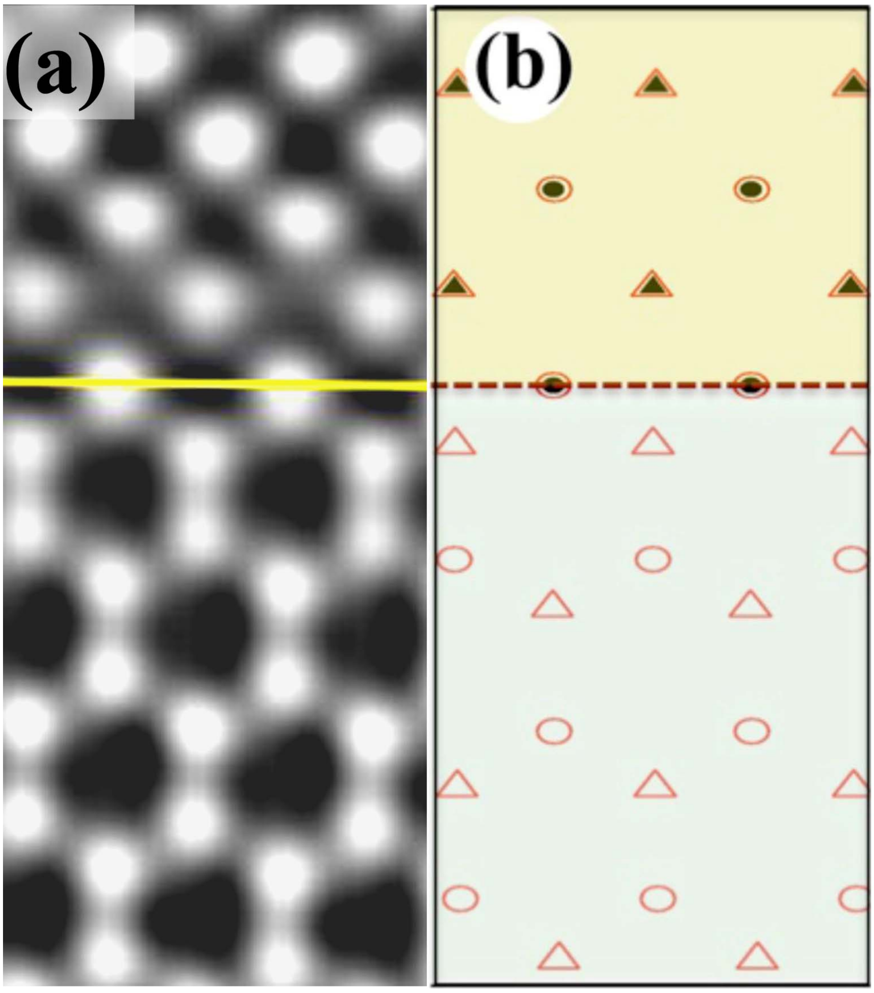

Figure S7. HRTEM analysis confirms the prediction of the stable phase interface. The yellow line in (a) is same as the brown dashed line in (b), indicating the shared atomic plane.
